# Assessing the progress on the implementation of policy and legislation actions to address the Non-Communicable Diseases crisis in the Pacific

**DOI:** 10.1371/journal.pone.0272424

**Published:** 2022-08-11

**Authors:** Si Thu Win Tin, Ilisapeci Kubuabola, Wendy Snowdon, Haley L. Cash, Elisiva Na’ati, Gade D. Waqa, Ada Moadsiri, Solene Bertrand, Amerita Ravuvu

**Affiliations:** 1 Public Health Division, Pacific Community (SPC), Suva, Fiji; 2 Division of Pacific Technical Support, World Health Organisation (WHO), Suva, Fiji; 3 Pacific Islands Health Officers’ Association (PIHOA), Honolulu, Hawaii; 4 Pacific Research Centre for the Prevention of Obesity and Non-Communicable Diseases (C-POND), College of Medicine, Nursing & Health Sciences, Fiji National University, Suva, Fiji; 5 Public Health, Division, Pacific Community (SPC), Noumea, New Caledonia; PLOS: Public Library of Science, UNITED KINGDOM

## Abstract

**Aim:**

To assess the progress on the implementation of Non-Communicable Diseases (NCD) related policies and legislations in the Pacific Island Countries and Territories (PICTs).

**Materials and methods:**

The Pacific Monitoring Alliance for NCD Action (MANA) Dashboard was used to assess the progress on the implementation. The MANA Dashboard includes 31 indicators across four different domains such as leadership and governance; preventative policies and legislations; health system response programs; and monitoring This progress assessment was conducted between 2019 and 2020 for all 21 PICTs. The data were analyzed and compared with the baseline status (2018) report and presented across four different domains of the MANA dashboard.

**Results:**

This progress assessment found that PICTs overall have made advancements in a number of areas, particularly the establishment of a national multi-sectoral NCD taskforce; implementation of referenced approaches to restrict trans-fat in the food supply in national documents; and fiscal measures to affect access and availability to less healthy foods and drinks. However, the strengths of actions varied across PICTs, and most are categorised as low strengths. Measures which had the most limited progress in implementation include policy and legislation that restrict alcohol advertising; tobacco industry interference; marketing of foods and non-alcoholic beverages to children; and marketing for breast milk substitutes.

**Conclusions:**

This progress assessment further highlights that while PICTs continue to make progress, NCD policy and legislation gaps still exist, both in terms of weaknesses of existing measures and areas that have had little attention to-date. These require urgent actions to scale up NCD related policies and legislation at regional and national level.

## 1. Introduction

Globally, the threats of Non-Communicable Diseases (NCD) to health and socioeconomic growth have been well recognised [1, 2]. Accounting for 75% of mortalities in the Pacific Island Countries and Territories (PICTs), NCD such as diabetes, cardiovascular diseases and chronic respiratory diseases and cancers are the Pacific’s top killer [3]. Premature deaths and disabilities from NCD have resulted in lost productivity that challenge the achievement of Sustainable Development Goals (SDGs) [4].

To strengthen multi-sectoral responses to the Pacific NCD crisis, in 2014, the Pacific leaders endorsed the Pacific NCD Roadmap and committed to implement NCD related policies and legislation [5] in line with global NCD best-buys [6]. Endorsed by the Pacific Health Ministers in 2017 [7], the Pacific Monitoring Alliance for NCD Action (MANA) Dashboard was used to identify the baseline implementation status (2018) [8]. This study aims to assess the progress on the implementation of NCD related policies and legislations in 2019–2020 in 21 PICTs, using the MANA Dashboard.

## 2. Materials and methods

This progress assessment using the MANA dashboard was conducted between 2019 and 2020 for all 21 PICTs. The MANA dashboard indicators and indicator ratings using a ‘traffic light’ scheme (Table 1) was used to assess the progress on the implementation of policies and legislation. The MANA Dashboard includes 31 indicators across four different domains such as leadership and governance; preventative policies and legislations; health system response programmes; and NCD monitoring [9, 10].

**Table 1:** Key for indicator ratings for the Pacific MANA dashboard.

Rating	Description
NA	Not applicable
	Not present
	Under development
	Present
Strength of action/implementation (star rating only assigned if ‘present’)	
**☆**	Low
**☆☆**	Medium
**☆☆☆**	High

PICTs that were assessed include American Samoa, the Commonwealth of the Northern Mariana Islands (CNMI), Cook Islands, Federated States of Micronesia (FSM), Fiji, French Polynesia, Guam, Kiribati, Nauru, Niue, New Caledonia, Palau, Papua New Guinea (PNG), Republic of the Marshall Islands (RMI), Tokelau, Tonga, Samoa, Solomon Islands, Tuvalu, Vanuatu, and Wallis and Futuna. The Pacific MANA coordination team members liaised with national NCD focal points in all 21 PICTs to update the MANA dashboards. The coordination team includes NCD policy experts from the Pacific Community (SPC), World Health Organization (WHO), the Pacific Islands Health Officers’ Association (PIHOA) and the Pacific Research Centre for Prevention of Obesity and Non-Communicable Diseases (C-POND) at the Fiji National University.

Members of the coordination team first updated the dashboard using publicly available information. The draft updated dashboard was distributed electronically via email to the respective national NCD focal person. The updated dashboards were reviewed and verified with supporting documentation and validated by national NCD focal persons and relevant national government authorities. The validated dashboards of all 21 PICTs have been endorsed by the Minister of Health or other appropriate signatory from each PICT. Given that FSM has national level and state level jurisdictions (Chuuk, Kosrae, Pohnpei, and Yap), the progress at the national level and in each state of the FSM were assessed and included in the analysis. If one state achieved a measure, it is counted as a progress made in the FSM.

The data of all 21 PICT dashboards were analysed and compiled in 2021 using Microsoft Excel 2016. The progress in 2019–2020 was compared and reported against the baseline implementation status (2018), as well as cumulative progress for all 21 PICTs.

## 3. Results

The following summarises the progress on the implementation across four different domains of the MANA dashboard.

### 3.1. Leadership and governance

Seven additional PICTs (CNMI, Cook Islands, Fiji, Kiribati, Nauru, RMI and Tokelau) that did not have a multisectoral NCD taskforce at baseline now have established taskforce in 2019–2020, resulting in a total of 12 PICTs establishing such a mechanism. Of these, only five PICTs (24%) (Guam, Kiribati, Palau, Samoa, and Tonga) were rated as having ‘strong’ (i.e., three stars green rating) functional taskforces. Additionally, four more PICTs (Nauru, New Caledonia, Solomon Islands and Tokelau) have developed a national multisectoral NCD strategy in 2019–2020, which brings the total to 17 PICTs. Of these, 11 PICTs (52%) were rated as having a ‘strong’ strategy in place. A few PICTs (FSM, French Polynesia and Palau) had further strengthened their NCD strategy and improved star ratings, while others like American Samoa had a NCD strategy which had expired at the time of reassessment and are in the process of developing a new one (Tables 2 and 3).

**Table 2:** PICT progress ratings on leadership and governance indicators (2018 vs. 2019–2020).

	American Samoa		Commonwealth of the Mariana Islands		Cook Islands		Federated States of Micronesia		Fiji		French Polynesia		Guam		Kiribati		Nauru		Niue	
Leadership and governance	B	P	B	P	B	P	B	P	B	P	B	P	B	P	B	P	B	P	B	P
L1. Multi-sectoral NCD taskforce				**☆**						**☆**			**☆☆☆**	**☆☆☆**		**☆☆☆**		**☆☆**		
L2. National strategy addressing NCDs and risk factors					**☆☆☆**	**☆☆☆**		**☆☆☆**	**☆☆☆**	**☆☆☆**		**☆☆**	**☆☆☆**	**☆☆☆**					**☆☆☆**	**☆☆☆**
L3. Explicit NCD indicators and targets	**☆☆**				**☆☆☆**	**☆☆☆**	**☆☆☆**	**☆☆☆**	**☆☆☆**	**☆☆☆**			**☆☆☆**	**☆☆☆**	**☆☆**	**☆☆**		**☆☆☆**		**☆**

B = Baseline 2018; P = Progress 2019–2020

**Table 3:** PICT progress ratings for leadership and governance indicators (2018 vs. 2019–2020).

	New Caledonia		Palau		PNG		Republic of the Marshall Islands		Samoa		Solomon Islands		Tokelau		Tonga		Tuvalu		Vanuatu		Wallis and Futuna	
Leadership and governance	B	P	B	P	B	P	B	P	B	P	B	P	B	P	B	P	B	P	B	P	B	P
L1. Multi-sectoral NCD taskforce			**☆☆☆**	**☆☆☆**				**☆**	**☆☆☆**	**☆☆☆**					**☆☆☆**	**☆☆☆**	**☆☆**	**☆☆**				
L2. National strategy addressing NCDs and risk factors		**☆**		**☆☆**	**☆☆☆**	**☆☆☆**			**☆☆☆**	**☆☆☆**		**☆☆☆**			**☆☆☆**	**☆☆☆**	**☆☆☆**	**☆☆☆**	**☆☆☆**	**☆☆☆**		
L3. Explicit NCD indicators and targets			**☆☆☆**	**☆☆☆**	**☆☆☆**	**☆☆☆**			**☆☆**	**☆☆☆**		**☆☆☆**	**☆☆☆**	**☆☆☆**	**☆☆☆**	**☆☆☆**	**☆☆**	**☆☆**	**☆☆☆**	**☆☆☆**		

B = Baseline 2018; P = Progress 2019–2020

### 3.2. Preventive policies and legislations

#### 3.2.1. Tobacco control

Three additional PICTs (RMI, Solomon Islands and Tokelau) that did not have tobacco taxation policies or had excise tax less than 20% of retail price, now reported having implemented new taxation measures in 2019–2020, resulting in 20 PICTs that have maintained and/or implemented fiscal measures on tobacco control. However, only six PICTs (29%) (American Samoa, French Polynesia, New Caledonia, Palau, Tonga, and Wallis & Futuna) were rated as having strong measures in place i.e., ‘three stars green rating’.

A total of 20 PICTs have a legislation to create smoke-free public places with two additional PICTs (FSM-Kosrae and Niue) now having new legislations in place. Niue and Samoa became the only PICTs which legislated measures to prevent tobacco industry interference. Two additional PICTs (American Samoa and Niue) have legislation requiring health warnings on tobacco packaging (total 17 PICTs); two additional countries (Niue, and Tokelau) have legislation to restrict advertising (total 19 PICTs); and four additional PICTs (FSM-Pohnpei, Niue, Samoa, and Tokelau) restrict sales and require tobacco licensing (total 18 PICTs). However, the strength of actions for tobacco control indicators varied greatly among countries (Tables 4 and 5).

**Table 4:** PICT progress ratings for preventive policy indicators (2018 vs. 2019–2020).

	American Samoa		Commonwealth of the Mariana Islands		Cook Islands		Federated States of Micronesia		Fiji		French Polynesia		Guam		Kiribati		Nauru		Niue	
Preventive policies	B	P	B	P	B	P	B	P	B	P	B	P	B	P	B	P	B	P	B	P
**Tobacco**																				
T1. Tobacco excise taxes	**☆☆**	**☆☆☆**	**☆☆**	**☆☆**	**☆☆**	**☆☆**	**☆**	**☆**	**☆**	**☆**	**☆☆☆**	**☆☆☆**	**☆**	**☆**	**☆**	**☆**	**☆**		**☆**	**☆**
T2. Smoke-free environments	**☆☆☆**	**☆☆☆**	**☆☆**	**☆☆**	**☆☆**	**☆☆☆**		**☆**	**☆**	**☆☆**	**☆☆☆**	**☆☆☆**	**☆☆☆**	**☆☆☆**	**☆☆**	**☆☆**	**☆☆☆**	**☆☆☆**		**☆☆☆**
T3. Tobacco health warnings					**☆☆**	**☆☆☆**			**☆☆☆**	**☆☆☆**	**☆☆☆**	**☆☆**	**☆**	**☆**						**☆☆☆**
T4. Tobacco advertising, promotion and sponsorship		**☆☆**				**☆☆☆**				**☆☆**	**☆☆☆**	**☆☆☆**			**☆☆**	**☆☆☆**	**☆☆☆**	**☆☆☆**		**☆☆☆**
T5. Tobacco sales and licencing	**☆☆☆**	**☆☆☆**	**☆☆☆**	**☆☆☆**	**☆**	**☆**		**☆**	**☆☆☆**	**☆☆**			**☆☆**	**☆☆**	**☆**	**☆**	**☆☆**	**☆☆**		**☆☆☆**
T6. Tobacco industry interference																				**☆☆☆**
**Alcohol**																				
A1. Alcohol licencing to restrict sales	**☆☆☆**	**☆☆☆**	**☆☆☆**	**☆☆☆**	**☆☆**	**☆☆**		**☆☆**	**☆☆**	**☆☆**	**☆☆**	**☆☆**	**☆☆☆**	**☆☆☆**	**☆☆**	**☆☆**	**☆☆**	**☆☆☆**	**☆☆**	**☆☆**
A2. Alcohol advertising								**☆☆☆**				**☆☆☆**								
A3. Alcohol taxation									**☆☆☆**	**☆☆☆**							**☆☆☆**	**☆☆☆**		
A4. Drink driving	**☆☆☆**	**☆☆**	**☆**	**☆**		**☆**			**☆☆**	**☆☆**	**☆☆**	**☆☆**	**☆☆**	**☆☆**	**☆**	**☆**	**☆☆**	**☆☆**	**☆**	**☆**
**Food**																				
F1. Reducing salt consumption					**☆☆**	**☆☆**	**☆☆☆**	**☆☆**	**☆☆☆**	**☆☆☆**	**☆☆**	**☆**	**☆☆**	**☆☆**	**☆☆**	**☆☆☆**	**☆**	**☆**		
F2. Trans-fats				**☆**		**☆**														
F3. Unhealthy food marketing to children						**☆☆☆**									**☆☆☆**	**☆☆☆**				
F4. Food fiscal policies					**☆**	**☆**	**☆**	**☆**	**☆**	**☆**	**☆**	**☆**			**☆☆☆**	**☆☆☆**	**☆☆☆**	**☆☆☆**	**☆☆**	**☆☆**
F5. Healthy food policies in schools	**☆☆**				**☆**	**☆**			**☆**	**☆**	**☆☆☆**	**☆☆☆**	**☆☆☆**	**☆☆☆**	**☆**	**☆**		**☆☆**	**☆☆**	**☆☆☆**
F6. Food-based dietary guidelines					**☆☆**	**☆☆**			**☆**	**☆**	**☆☆**	**☆☆**	**☆☆**	**☆☆**	**☆☆☆**	**☆☆☆**			**☆☆☆**	**☆☆☆**
**Physical Activity**																				
P1. Compulsory physical education in school curriculum	**☆☆☆**			**☆☆☆**	**☆☆**	**☆☆☆**					**☆☆☆**	**☆☆☆**	**☆**	**☆**	**☆☆☆**	**☆☆☆**	**☆☆☆**	**☆☆☆**	**☆☆☆**	**☆☆☆**
**Enforcement**																				
E1. Enforcement of laws and regulations related to NCD risk factors		**☆**	**☆☆☆**	**☆☆**		**☆☆☆**		**☆**					**☆**	**☆**	**☆**	**☆☆☆**				

B = Baseline 2018; P = Progress 2019–2020

**Table 5:** PICT progress ratings for preventive policy indicators (2018 vs. 2019–2020).

	New Caledonia		Palau		PNG		Republic of the Marshall Islands		Samoa		Solomon Islands		Tokelau		Tonga		Tuvalu		Vanuatu		Wallis and Futuna	
Preventive policies	B	P	B	P	B	P	B	P	B	P	B	P	B	P	B	P	B	P	B	P	B	P
**Tobacco**																						
T1. Tobacco excise taxes	**☆☆☆**	**☆☆☆**	**☆☆☆**	**☆☆☆**		**☆☆**		**☆**	**☆☆**	**☆☆**					**☆☆☆**	**☆☆☆**	**☆☆**	**☆**	**☆**	**☆**	**☆☆☆**	**☆☆☆**
T2. Smoke-free environments	**☆☆☆**	**☆☆☆**	**☆☆**	**☆☆**	**☆☆☆**	**☆☆☆**	**☆☆**	**☆☆**	**☆☆**	**☆☆**	**☆**	**☆**	**☆☆**	**☆☆**	**☆☆**	**☆☆**	**☆☆☆**	**☆☆**	**☆☆☆**	**☆☆☆**		
T3. Tobacco health warnings	**☆**	**☆☆**			**☆☆**	**☆☆☆**			**☆☆**	**☆☆☆**	**☆☆**	**☆☆**			**☆☆**	**☆☆**	**☆**		**☆☆☆**	**☆☆**		
T4. Tobacco advertising, promotion and sponsorship	**☆☆☆**	**☆☆☆**	**☆☆☆**	**☆☆☆**	**☆☆**	**☆☆**	**☆☆**		**☆☆☆**	**☆☆**				**☆**	**☆☆**	**☆☆**	**☆☆☆**	**☆☆**	**☆☆**	**☆☆☆**	**☆☆☆**	**☆☆☆**
T5. Tobacco sales and licencing			**☆☆☆**	**☆☆☆**	**☆☆☆**	**☆☆**		**☆**		**☆☆☆**	**☆☆☆**	**☆☆☆**			**☆**	**☆**	**☆☆☆**	**☆☆☆**	**☆☆☆**	**☆☆☆**		
T6. Tobacco industry interference										**☆☆☆**												
**Alcohol**																						
A1. Alcohol licencing to restrict sales	**☆☆☆**	**☆☆**	**☆☆☆**	**☆☆☆**	**☆☆**	**☆☆☆**	**☆☆**	**☆☆**	**☆☆**	**☆☆☆**	**☆☆☆**	**☆☆☆**	**☆☆**	**☆☆**	**☆☆☆**	**☆☆☆**	**☆☆☆**	**☆☆☆**	**☆☆**	**☆☆**	**☆☆**	**☆☆**
A2. Alcohol advertising	**☆☆**	**☆☆☆**																				
A3. Alcohol taxation	**☆☆☆**	**☆☆☆**				**☆☆**			**☆**	**☆**	**☆☆**	**☆☆**				**☆☆**	**☆☆☆**	**☆☆**				
A4. Drink driving	**☆☆**	**☆☆☆**	**☆☆**	**☆☆**			**☆**	**☆**		**☆**	**☆☆**	**☆☆**			**☆☆**	**☆☆**	**☆**	**☆**			**☆☆☆**	**☆☆**
**Food**																						
F1. Reducing salt consumption	**☆☆**	**☆☆**	**☆**	**☆**	**☆**				**☆☆☆**	**☆☆☆**		**☆**				**☆**		**☆**				
F2. Trans-fats										**☆☆**								**☆**				
F3. Unhealthy food marketing to children										**☆**												
F4. Food fiscal policies		**☆**					**☆**	**☆**	**☆☆☆**	**☆☆☆**			**☆☆☆**		**☆☆☆**	**☆☆☆**			**☆☆**	**☆☆**	**☆**	**☆**
F5. Healthy food policies in schools	**☆**	**☆**		**☆☆**					**☆**	**☆☆☆**						**☆☆☆**						
F6. Food-based dietary guidelines	**☆☆**	**☆☆☆**		**☆☆**			**☆**		**☆☆☆**	**☆☆☆**	**☆☆☆**	**☆☆☆**			**☆☆**			**☆☆☆**				
**Physical Activity**																						
P1. Compulsory physical education in school curriculum		**☆☆**			**☆☆**	**☆☆☆**			**☆☆**	**☆☆☆**	**☆☆**	**☆☆**	**☆☆**	**☆☆**	**☆**						**☆☆☆**	**☆☆☆**
**Enforcement**																						
E1. Enforcement of laws and regulations related to NCD risk factors				**☆**						**☆☆**	**☆**				**☆**	**☆☆☆**	**☆☆**	**☆☆**			**☆☆☆**	**☆☆☆**

B = Baseline 2018; P = Progress 2019–2020

#### 3.2.2. Alcohol control

One additional country (RMI) has implemented alcohol taxation measures resulting in 21 PICTs that have maintained and/or implemented alcohol taxation measures, but in most cases, taxation is based on beverage type rather than on ethanol content, and only three PICTs (14%) (Fiji, Nauru, and New Caledonia) have ‘strong’ measures in place. All 21 PICTs have national licensing regulations in place to restrict the sale of alcohol, two additional PICTs (FSM-Pohnpei and PNG) have regulations in place to prevent drink driving (total 20 PICTs), and two additional PICTs (FSM and Nauru) restrict alcohol advertising (total 8 PICTs). The strength of actions for alcohol control indicators varied greatly among countries, and most countries have low strength of actions on advertising, taxation, and drink driving (Tables 4 and 5).

#### 3.2.3. Food and physical activity

One additional country (New Caledonia) adopted a taxation measure to discourage unhealthy food/beverage choices, such as taxation on sugar sweetened beverages (SSB) and unhealthy foods, bringing the total to 14 PICTs. However only four PICTs (19%) (Kiribati, Nauru, Samoa, and Tonga) were rated as having ‘strong’ measures in place.

One additional country (Tonga) put in place a policy to reduce population salt consumption resulting in 16 PICTs in total; and six additional PICTs (American Samoa, CNMI, Cook Islands, Niue, Samoa, and Tuvalu) have referenced approaches to restrict trans-fat in the food supply in national documents (from none to now six PICTs in total). However, the strengths of actions are low (no or one-star green rating) in most PICTs.

Two additional PICTs (Palau and Tuvalu) have endorsed food based dietary guidelines resulting in 13 PICTs in total; three additional PICTs (Cook Islands, Niue, and Samoa) have put in place policies to restrict marketing of foods and non-alcoholic beverages to children resulting in five PICTs in total, with four more PICTs (Nauru, Palau, PNG, and Tonga) putting in place policies to encourage the provision and promotion of healthy food choices in schools, which now brings the total to 14 PICTs. Two additional PICTs (CNMI and Palau) have compulsory physical education in school (total 15 PICTs). While some PICTs have strong actions that address food and physical activity policy, many were still rated as being of low strength (Tables 4 and 5).

#### 3.2.4. Enforcement

Five additional PICTs (American Samoa, FSM-Pohnpei, Palau, RMI and Tokelau) have a national level system in place to support enforcement of NCD risk factors however the strengths of most enforcement systems were weak with only four PICTs (19%) (Cook Islands, Kiribati, Tonga and Wallis and Futuna) rated as having strong systems in place (Tables 4 and 5).

Some PICTs had further strengthened preventive policies and legislations on tobacco, alcohol and less healthy diets, and improved star ratings overall. However, it was found that ratings of some baseline indicators in a few PICTs were not accurate and therefore readjusted to a lower rating in the updated dashboards e.g., food policy and physical education curriculum indicators in American Samoa and enforcement indicators for Fiji and the Solomon Islands (Tables 4 and 5).

### 3.3. Health system response programmes

One additional PICT (Palau) has national guidelines in place for the diagnosis and management of at least one of the four main NCDs (total 19 PICTs) and four additional PICTs (FSM-Pohnpei, PNG, RMI, and Solomon Islands) have all identified essential NCD medicines included in the national list of essential medicines (total 19 PICTs). Four additional PICTs (FSM, PNG, Tokelau, and Wallis & Futuna) have smoking cessation support of some kind available (total 18 PICTs).

Regarding programs related to infant nutrition, one additional PICT (Samoa) has restrictions on the marketing of breast milk substitutes (total six PICTs), one additional PICT (PNG) has a public hospital certified as a baby-friendly hospital (total five PICTs), and six additional PICTs (CNMI, Guam, Samoa, Tuvalu, and Wallis & Futuna) have legislation to provide at least 12 weeks paid maternity leave and breast-feeding facilities, resulting in a total of 14 PICTs. The strengths of actions for health system response programmes varied across PICTs (Tables 6 and 7).

**Table 6:** PICT progress ratings for health system response indicators (2018 vs. 2019–2020).

	American Samoa		Commonwealth of the Mariana Islands		Cook Islands		Federated States of Micronesia		Fiji		French Polynesia		Guam		Kiribati		Nauru		Niue	
Health system response programmes	B	P	B	P	B	P	B	P	B	P	B	P	B	P	B	P	B	P	B	P
H1. National guidelines for care of main NCDs	**☆☆**	**☆**			**☆**	**☆☆☆**		**☆**	**☆**	**☆**	**☆☆☆**		**☆☆**	**☆☆**	**☆**	**☆**	**☆**	**☆☆**	**☆☆☆**	**☆☆**
H2. Essential drugs		**☆☆☆**	**☆☆☆**			**☆☆☆**		**☆☆**	**☆**	**☆**	**☆☆☆**	**☆☆☆**		**☆☆☆**	**☆☆**	**☆☆**	**☆☆**	**☆☆☆**	**☆☆**	
H3. Smoking cessation	**☆**	**☆☆**	**☆☆**	**☆☆**	**☆☆**	**☆☆**			**☆**	**☆**	**☆☆**	**☆☆☆**	**☆☆**	**☆☆☆**					**☆☆☆**	**☆☆☆**
H4. Marketing of breast milk substitutes									**☆☆☆**	**☆☆**						**☆☆**				
H5. Baby friendly hospitals									**☆**	**☆**										
H6. Maternity leave and breastfeeding		**☆**		**☆**					**☆**	**☆**	**☆☆☆**	**☆☆☆**		**☆☆☆**	**☆**	**☆**				

B = Baseline 2018; P = Progress 2019–2020

**Table 7:** PICT progress ratings for health system response indicators (2018 vs. 2019–2020).

	New Caledonia		Palau		PNG		Republic of the Marshall Islands		Samoa		Solomon Islands		Tokelau		Tonga		Tuvalu		Vanuatu		Wallis and Futuna	
Health system response programmes	B	P	B	P	B	P	B	P	B	P	B	P	B	P	B	P	B	P	B	P	B	P
H1. National guidelines for care of main NCDs	**☆☆☆**	**☆☆☆**		**☆☆**	**☆**	**☆**			**☆**	**☆☆**	**☆☆☆**	**☆☆☆**	**☆☆**	**☆☆**	**☆☆☆**	**☆☆☆**	**☆☆**	**☆☆**	**☆**	**☆**	**☆☆☆**	**☆☆☆**
H2. Essential drugs	**☆☆☆**	**☆☆☆**							**☆☆**	**☆☆☆**			**☆☆☆**	**☆☆☆**	**☆☆☆**	**☆☆☆**	**☆☆**	**☆☆**			**☆☆☆**	**☆☆☆**
H3. Smoking cessation	**☆☆☆**	**☆☆☆**	**☆**	**☆☆**		**☆**					**☆☆**	**☆☆**			**☆☆☆**	**☆☆☆**			**☆**	**☆**		
H4. Marketing of breast milk substitutes			**☆☆☆**	**☆☆☆**																		
H5. Baby friendly hospitals																						
H6. Maternity leave and breastfeeding		**☆☆☆**								**☆☆☆**												

B = Baseline 2018; P = Progress 2019–2020

### 3.4. Monitoring

The collection of new data for adult NCD risk prevalence has been completed in two additional PICTs (RMI and Wallis & Futuna) (total 14 PICTs) while three additional PICTs (American Samoa, Niue, and RMI) have undertaken monitoring of adolescent prevalence data (total 17 PICTs). With one additional PICT (PNG), 19 PICTs now have functioning systems for generating cause-specific mortality data on a routine basis. Four additional PICTs (New Caledonia, RMI, Tonga, and Wallis & Futuna) now routinely collect and report child growth data, therefore bringing the total to 15 PICTs. However, the strength of actions for monitoring indicators varied greatly among countries (Tables 8 and 9).

**Table 8:** PICT progress ratings for monitoring indicators (2018 vs. 2019–2020).

	American Samoa		Commonwealth of the Mariana Islands		Cook Islands		Federated States of Micronesia		Fiji		French Polynesia		Guam		Kiribati		Nauru		Niue	
Monitoring	B	P	B	P	B	P	B	P	B	P	B	P	B	P	B	P	B	P	B	P
M1. Population risk factor prevalence surveys—adults	**☆☆**	**☆☆**	**☆☆**	**☆☆**	**☆☆☆**	**☆☆☆**	**☆☆☆**		**☆**	**☆**			**☆**	**☆☆**	**☆☆**	**☆☆☆**	**☆☆**	**☆☆**		
M2. Population risk factor prevalence surveys—youth					**☆☆☆**	**☆**	**☆☆☆**	**☆☆☆**	**☆**	**☆**	**☆☆☆**	**☆☆☆**								**☆☆☆**
M3. Child growth monitoring			**☆**	**☆**	**☆☆**	**☆☆☆**											**☆☆☆**	**☆☆☆**	**☆☆☆**	**☆☆☆**
M4. Routine cause-specific mortality	**☆☆☆**		**☆☆☆**	**☆☆☆**	**☆☆☆**	**☆☆☆**	**☆☆**	**☆☆**	**☆**	**☆**	**☆☆☆**	**☆☆**	**☆**	**☆**	**☆☆☆**	**☆☆☆**	**☆☆☆**	**☆☆☆**	**☆☆☆**	**☆☆☆**

B = Baseline 2018; P = Progress 2019–2020

**Table 9:** PICT progress ratings for monitoring indicators (2018 vs. 2019–2020).

	New Caledonia		Palau		PNG		Republic of the Marshall Islands		Samoa		Solomon Islands		Tokelau		Tonga		Tuvalu		Vanuatu		Wallis and Futuna	
Monitoring	B	P	B	P	B	P	B	P	B	P	B	P	B	P	B	P	B	P	B	P	B	P
M1. Population risk factor prevalence surveys—adults	**☆☆☆**		**☆☆**	**☆☆**	**☆**			**☆☆**	**☆☆☆**		**☆☆**	**☆☆**	**☆☆☆**	**☆☆☆**	**☆☆☆**	**☆☆☆**	**☆☆☆**	**☆☆☆**				**☆☆**
M2. Population risk factor prevalence surveys—youth			**☆☆☆**	**☆☆☆**			**☆**	**☆**		**☆☆☆**			**☆☆☆**	**☆☆☆**	**☆☆☆**	**☆☆☆**	**☆☆☆**	**☆☆**	**☆☆☆**	**☆☆☆**	**☆☆☆**	**☆☆☆**
M3. Child growth monitoring			**☆☆☆**	**☆**	**☆☆☆**	**☆☆**		**☆☆☆**					**☆**	**☆**		**☆**	**☆☆**	**☆☆**				
M4. Routine cause-specific mortality	**☆☆☆**	**☆☆☆**	**☆☆☆**	**☆☆**			**☆☆**	**☆☆**	**☆☆☆**	**☆☆☆**					**☆☆**	**☆☆**	**☆☆☆**	**☆☆☆**				

B = Baseline 2018; P = Progress 2019–2020

## 4. Discussion and conclusion

This progress assessment found that PICTs have made advancements in a number of areas, particularly the establishment of a national multi-sectoral NCD taskforce; implementation of referenced approaches to restrict trans-fat in the food supply in national documents; measures to provide at least 12 weeks paid maternity leave; and taxation-based approaches and measures to affect access and availability to less healthy foods and drinks. However, the strengths of actions varied across PICTs, and most are categorised as low strength. Limited progress was seen in the implementation of policy or legislation to restrict alcohol advertising; tobacco industry interference; marketing of foods and non-alcoholic beverages to children; marketing of breast milk substitutes; and establishing baby friendly hospitals.

Given that political leaders have the power to bring stakeholders in all relevant sectors together, it is critical to make NCD a politically relevant issue and that political leaders make NCD a priority. The leadership role of politicians are critical to ensure that countries have national multisectoral NCD strategies and functioning multisectoral NCD taskforces, as these are the essential components to enhance multisectoral actions [11]. This study has shown that having a strong governing mechanism at the national level has the potential for more improved NCD actions. For example, PICTs such as Guam, Samoa, and Tonga all have a well-established and functioning multi-sectoral taskforce (green with three stars rating) and are operative and advanced in taking NCD actions when compared to the PICTs where such mechanisms are not yet in place. Despite improvements made in the leadership and governance areas, there is still a significant challenge to strengthen and sustain existing efforts. The COVID-19 pandemic, natural disasters, divergent and conflicting mandates of government ministries, and other competing priorities continue to challenge sustaining leadership and commitment on NCD actions [12].

The legal implications of regulating tobacco, alcohol and less healthy foods remain a challenge to navigate [13]. In many countries, significant efforts are still needed to regulate these risk factors. Sustained political will and collective societal commitments are needed to strengthen these. For the Pacific region, it has struggled to optimise legislative measures to incentivise health goals [14]. With tobacco control measures, the majority of PICTs have made significant strides [15], however enforcement remains a gap. A major obstacle to implementation of effective tobacco control laws is the interference of the tobacco industry. For most PICTs, preventing tobacco industry interference remains an undeveloped and/or under development area. Only two countries across the region—Samoa and Niue, have a policy that protects government from the influences of the tobacco industry. The tobacco industry continues to find ways to maintain and expand its interests globally [16], and there is a critical need to recognise tobacco industry interference and its pervasiveness throughout the region.

In terms of raising taxes on tobacco products, while most PICTs have used tax and price measures to reduce the demand for tobacco, only six countries have raised tobacco excise taxes to the recommended global benchmark of more than 70% of the retail price [17, 18]. The implementation of effective measures to ensure that tobacco product packaging and labelling, in line with Article 11 of the WHO Framework Convention on Tobacco Control (FCTC), still poses a challenge in most PICTs. The drawback here can be largely attributed to limited capacity and resourcing in PICTs and lack of priority placed by leaders. PICTs have been active in strengthening their smoke-free legislation, but there are opportunities to expand the number of smoke-free places covered in legislation. Regarding tobacco advertising, promotion and sponsorship, the majority of PICTs have full or partial bans, but the use of mass media needs to be capitalised in PICTs to raise public awareness of the harms caused by tobacco use and exposure to tobacco smoke. There is also an opportunity for greater engagement with civil society playing a more active role in raising awareness and conducting advocacy campaigns to compliment tobacco control legislations.

Alcohol use remains a leading risk factor for disease and injury in PICTs [19, 20] and legislations to regulate its harmful use and consumption vary across PICTs. With respect to alcohol tax policy, all PICTs have an alcohol excise taxation system in place but given that taxation is based on beverage type rather than on ethanol content for the majority, this can be argued to be regressive, weighing more heavily on poor drinkers than it does on rich drinkers [21]. Furthermore, taxes that reflect pure alcohol content are argued to have more of a proportional effect on beer, wine, and spirits [22]. The results also report that only Fiji, Nauru and New Caledonia have strong measures in place i.e., having an excise tax based on ethanol content that is adjusted annually for inflation, and has an alcohol taxation policy based on an explicit statement by government. It is critical that PICT governments are clear about the primary goal of their alcohol taxes and that they frame the tax accordingly, as not doing so leaves such fiscal measures vulnerable to hostile lobbying [23]. In terms of licensing, it is encouraging to note that all PICTs have licensing regulations in place to restrict the sale of alcohol but there are opportunities to strengthen licensing controls.

With regards to alcohol advertising, the results clearly show that regulation remains weak across the region. Consequently, alcohol products are freely advertised and aggressively marketed. Much of what was found in a 2006 Massey University report [24] remains unchanged for this indicator. For instance, Tonga has no alcohol marketing regulations in place to date. For the Cook Islands, a 1998 Health Islands Committee recommended against all forms of alcohol advertising and sponsorship, but this is an area that remains under development. In Fiji and Guam, alcohol advertising is still heavily advertised and marketed with an absence of an alcohol marketing regulation to date. Regulating alcohol advertising is an area that many PICTs struggle with and globally, this is an area where considerable debate around the appropriate policy response [25].

Evidently, policy and legislation interventions significantly contribute to making diets healthier and prevent NCD. In terms of food fiscal policies, the Pacific region is an example to other regions, particularly with the widespread use of sugar sweetened beverages (SSBs) taxes and the high rates of many of these taxes [26]. Many PICTs have adopted a taxation measure on SSBs. However, there are opportunities for PICTs to pursue taxes on other less healthy food products as well as part of a suite of measures to improve diets. PICTs have also been proactive in adopting a range of food policies and regulations to improve diets including regulations on compositional standards for salt and sugar in foods or school food policies [27, 28] through the adoption and adaptation of a food based dietary guidelines. However, effective implementation is a challenge that will need to be sustained in these areas to enhance health outcomes.

Despite some progress, significant barriers stand in the way of development of trans-fat regulations, and most PICTs have yet to develop trans-fat policies and regulations that limit or eliminate industrially produced trans-fatty acids from their food supply. Similarly, unhealthy food marketing to children is an area that requires attention across the region as this remains largely undeveloped. A multi-sectoral collaboration approach with polices to guide and legislations to regulate will progress efforts to improve diet. Breastmilk provides optimal nutrition for infants in the first six months of life which is critical for laying the foundation for lifelong health and development and reduces the risk of NCD later in life. Programmes and initiatives to promote breastfeeding serve as enablers for good dietary practice and healthy eating for young children. However, the findings show most PICTs lack regulations to restrict marketing of breastmilk substitutes and for those that have, the regulations are not aligned with the International Code of marketing of breastmilk substitutes or not enforced.

Schools play an important role to support children to learn and practice lifelong healthy behaviours and strong commitment from policy leaders is needed to create healthy environment within schools [29]. The findings also demonstrate that physical education in school curriculum and implementation of the syllabus has become mandatory and enforced in 15 PICTs. This demonstrates the improved collaborative efforts of health and education ministries in investing and supporting required resources in schools. Behaviours learnt in school become habitual as children grow to positively influence families, communities, and societies. Positive attitude towards a healthy lifestyle behaviour during childhood will create youth empowerment and leadership for health in the future.

Despite some progress seen in the enforcement of laws and regulations related to NCD risk factors, the strength of actions still needs to be enhanced. There is much evidence to support that robust public health policy and legislation is key to the delivery of effective public health actions, however many PICTs still have limited capacity and expertise to implement and enforce the recommended NCD related laws. Given that government regulation of NCD is multisectoral and different government authorities deal with different NCD related laws, it increases the challenges associated with the effective implementation and enforcement of NCD related laws. An integrated government approach and commitment from political leaders is critical to ensure that enforcement is in place and actively pursued at the national level.

With regards to NCD management, most countries have national guidelines in place and essential NCD medications are included in the national essential drugs list. However, it is important that NCD medications are continuously available without stock-outs which is a common challenge in PICTs. With the COVID-19 pandemic, despite evidence of greater risks from the effects of COVID-19 in those with NCD, PICTs face challenges specifically in the disruption of NCD management due to the postponement of screening programs, cancellation of planned treatments and shortage of essential medicines. This exacerbates the ongoing challenges that PICTs face in addressing the existing Pacific NCD crisis [12], and will remain off-track to achieving global NCD targets particularly to reduce premature mortality from NCD [30].

In terms of NCD related population-based monitoring, most PICTs have completed adult and adolescent NCD risk factor surveys. However, further action is still required to ensure that surveys are scheduled regularly and that the findings are used for decision- making and planning to guide further interventions. The challenge in PICTs is that a lot of data are collected but never reported or reported after long delays that make them challenging to use for planning. In addition, mortality data are often incomplete and poor quality highlighting the need to strengthen Civil Registration and Vital Statistics (CRVS) systems. There is also a need to enhance child growth monitoring for effective interventions that address the double burden of malnutrition in PICTs.

Although the assessment identified the progress made, there are some limitations. PICTs started and completed updating their dashboards at different times, and the validated dashboards were endorsed by the appropriate signatories from each PICT at different timeframes in 2019–2020. Therefore, the progress made since the endorsement of PICTs’ updated MANA dashboards may not be reflected in this paper. The endorsement process took longer than expected in some countries because of the COVID-19 pandemic and competing priorities at the national level. However, the strength of the assessment was the consistent use of indicator criteria and definitions, and indicator ratings for both the baseline and follow-up assessments, making comparisons and assessing the progress reliable and credible. As this was the second round of data collection using the same methodology, it was less time-consuming than the baseline data collection and validation. When required, the dashboard indicators will be subject to further refinement to accommodate potential drivers of change that challenge and influence the prevention and control of NCD across the Pacific.

In conclusion, this progress assessment using the MANA dashboard mutual accountability mechanism further highlights that while PICTs continue to make progress, NCD policy gaps still exist, both in terms of weaknesses of existing measures and areas that have had little attention to-date. These require urgent actions at the regional and national level. Political, environmental and socioeconomic factors continue to hamper progress on all aspects of NCD prevention and control across the Pacific. Investing additional resources and scaling up actions on NCD related policies and legislations will ensure the prevention of NCD, safeguarding of people with NCD, minimising co-morbidity due to emerging and re-emerging infectious diseases, and promoting the well-being of all Pacific people. This will contribute to meeting the global NCD targets and SDGs particularly to reduce premature mortality from NCD by one third by 2030, and to achieve the Pacific’s Healthy Island Vision.
